# A service-based approach to cryoEM facility processing pipelines at eBIC

**DOI:** 10.1107/S2059798324000986

**Published:** 2024-02-20

**Authors:** Anna Horstmann, Stephen Riggs, Yuriy Chaban, Daniel K. Clare, Guilherme de Freitas, David Farmer, Andrew Howe, Kyle L. Morris, Daniel Hatton

**Affiliations:** a Data Analysis, Diamond Light Source, Harwell Science and Innovation Campus, Didcot OX11 0DE, United Kingdom; b Electron Bio-Imaging Centre (eBIC), Diamond Light Source, Harwell Science and Innovation Campus, Didcot OX11 0DE, United Kingdom; c Scientific Information Management Systems, Diamond Light Source, Harwell Science and Innovation Campus, Didcot OX11 0DE, United Kingdom; d Dubochet Centre for Imaging, University of Geneva, Quai Ernest-Ansermet 30, Geneva, Switzerland; e EMDB (Electron Microscopy Data Bank), European Molecular Biology Laboratory, European Bioinformatics Institute, Wellcome Genome Campus, Hinxton, Cambridge CB10 1SD, United Kingdom; Rutherford Appleton Laboratory, United Kingdom

**Keywords:** cryoEM facilities, eBIC, processing pipelines, cloud computing

## Abstract

Electron cryo-microscopy image-processing workflows are typically composed of elements that may, broadly speaking, be categorized as high-throughput workloads which transition to high-performance workloads as preprocessed data are aggregated. The high-throughput elements are of particular importance in the context of live processing, where an optimal response is highly coupled to the temporal profile of the data collection. In other words, each movie should be processed as quickly as possible at the earliest opportunity. The high level of disconnected parallelization in the high-throughput problem directly allows a completely scalable solution across a distributed computer system, with the only technical obstacle being an efficient and reliable implementation. The cloud computing frameworks primarily developed for the deployment of high-availability web applications provide an environment with a number of appealing features for such high-throughput processing tasks. Here, an implementation of an early-stage processing pipeline for electron cryotomography experiments using a service-based architecture deployed on a Kubernetes cluster is discussed in order to demonstrate the benefits of this approach and how it may be extended to scenarios of considerably increased complexity.

## Abstract 2

The automation of cryoEM pipelines to aid data-quality analysis during acquisition poses a number of challenges, particularly at facilities where the expected data rates are high. The use of a modern service-based architecture deployed on Diamond Light Source’s on-premises cloud computing cluster to tackle some of these challenges is demonstrated, focusing on the provision of an early-stage processing pipeline for electron tomography that produces first-attempt reconstructed volumes within minutes of tilt-series acquisition.

## 1. Introduction

Data-quality assessment for electron cryo-microscopy (cryoEM) often requires some amount of image processing following the acquisition process. For facilities, which provide limited microscope time for large numbers of users, it is advantageous to perform automated analysis for this purpose. Automation allows users to focus on microscope operations, possibly in response to the processing results. CryoEM processing involves a wide range of applications, with a correspondingly wide range in resource requirements. Typically, cryoEM processing pipelines transition from a high-throughput environment, where relatively small tasks can be completely parallelized, to an environment closer to high-performance computing, where large computing resources need to be focused on a single task. This requires an organization of resources that is, ideally, dynamic and robust, with a simple path to scaling should demands increase either through an expansion of the facility or software-enabled increases in data-collection rates. Facility processing pipelines have been addressed elsewhere, such as in *Scipion* (de la Rosa-Trevín *et al.*, 2016 ▸) and *Appion* (Lander *et al.*, 2009 ▸). Here, we focus on scalable deployments using modern tools developed for cloud computing environments.

These abstracted challenges have been addressed in a variety of ways in enterprise computing. Here, we demonstrate the use of the container orchestration system Kubernetes (https://kubernetes.io/), which has found wide application in cloud computing platforms, to deploy automated cryoEM processing pipelines meeting the requirements of large-scale cryoEM facilities. In particular, we examine an early-stage electron cryotomography (cryoET) pipeline currently deployed at the electron Bio-Imaging Centre (eBIC) at Diamond Light Source (Clare *et al.*, 2017 ▸). We make use of the Zocalo processing framework (Gerstel *et al.*, 2019 ▸), developed at Diamond Light Source, to organize parts of the workflow and introduce a new tool to manage data transfer from microscope and detector systems to a facility filesystem for processing. Processing requests are directly connected to the successful completion of data transfer through the same tool, minimizing the time between data accessibility and processing.

In Section 2 we describe the basics of the early-stage processing pipeline and the cryoEM software components used. This leads to a more general discussion of the structure of processing pipelines and their representation. In Section 3 we lay out the technical requirements of the implementation of this pipeline from the facility perspective. Section 4 gives very brief descriptions of the technologies that we leverage in our implementation, while Section 5 outlines how these technologies are mapped onto the representation described in Section 2. The connection between processing and data acquisition is then detailed, followed by discussion of the usage of the pipeline to date, both in the user program and internal research, and the presentation of a web-based application for visualization. Appendix *A* [App appa] provides some statistics indicating the cryoET pipeline utilization for 2023 at eBIC.

## 2. Tomography early-stage processing pipeline

To provide an early means of quality assessment without the complication of sample-dependent processing decisions, we aim only to produce first-attempt reconstructions from each tilt series collected. We also execute CTF estimation as this is likely to be required in downstream processing. There are therefore three steps we need to consider: (i) motion correction of each tilt, (ii) CTF estimation of each motion-corrected micrograph and (iii) alignment and reconstruction of each tilt series, operating on the stack of motion-corrected micrographs corresponding to that tilt series. In addition, for the purposes of improved visibility of the feature of interest we can consider a fourth step: (iv) tomogram denoising.

Alignment and reconstruction are considered as a single step as they are performed using the same piece of software. We use *MotionCor*2 for motion correction (Zheng *et al.*, 2017 ▸), *CTFFind*4 (Rohou & Grigorieff, 2015 ▸) for CTF estimation and *AreTomo* (Zheng *et al.*, 2022 ▸) for alignment and reconstruction, allowing the automated reconstruction of volumes without fiducials. Basic tomogram denoising is performed with *Topaz* (Bepler *et al.*, 2020 ▸) using a pre-trained model to avoid user input for training. We also use functionality in *IMOD* (Kremer *et al.*, 1996 ▸) to perform basic manipulation of the micrographs.

### 2.1. Processing-pipeline representation

**Figure 1:**
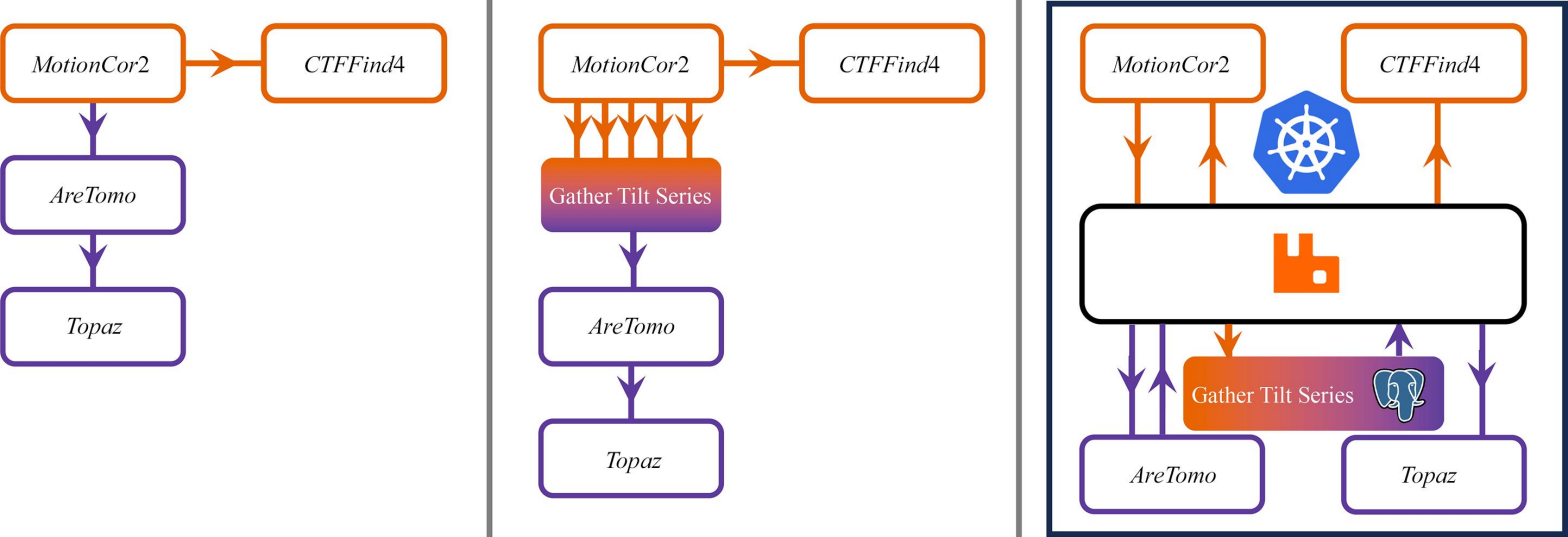
The first panel shows a simple graphical representation of an early-stage cryoET pipeline. Every movie in a tilt series must be motion-corrected before performing CTF estimation on the resulting micrograph. The motion-corrected micrographs for each tilt in a tilt series are combined together for alignment and tomographic reconstruction. This combination is represented in the second panel as a node with multiple inputs mapping to a single reconstruction processing request. The third panel partially details how this representation is mapped to a variety of technologies, as outlined in Section 5. The box labelled ‘Gather Tilt Series’ is implemented as a server making use of a PostgreSQL database, as mentioned at the end of Section 6.

Typically, data-processing pipelines or workflows are represented as directed acyclic graphs (DAGs). This is common to a variety of workflow frameworks such as Apache Airflow, DAGMan, Dask and Zocalo. In the domain of cryoEM processing, *RELION* (Scheres, 2012 ▸) models input and output files as vertices in a DAG along with the job types (Fernandez-Leiro & Scheres, 2017 ▸), with edges providing the distinction between input and output. These cases differ in the details of how the representation is constructed, as there is significant ambiguity in the mapping of the implemented components of a given processing pipeline onto the sets of vertices and edges that form the graph. One simple representation uses a graph to indicate the dependency of processing steps on one another, as depicted in the first panel of Fig. 1 ▸. This does not adequately represent that all tilts in a tilt series must be motion-corrected before the reconstruction of the corresponding tomogram can be performed. The transition from operations performed on individual movies/micrographs to composite data sets such as complete tilt series is difficult to represent inside a single DAG workflow. This step is represented in the second panel of Fig. 1 ▸ as a vertex with multiple input edges but only a single output. We separate out the stages acting on different data domains (represented with different colours in Fig. 1 ▸) into different processing ‘recipes’ in Zocalo and use a database to register information regarding which tilt images have been motion-corrected so that the reconstruction can be requested at the appropriate time.

Our aim is to construct a practical system onto which we can map these kinds of graphical representations. This involves a concrete choice of technologies. (The third panel of Fig. 1 ▸ depicts a simplified version of the specific implementation discussed further in Section 5.) To inform such choices, we provide some technical requirements of the system given the context of a cryoEM facility.

## 3. Technical requirements

The processing needs of scientific facilities require large and often varied computational resources. The necessity of applying distributed computing for large-scale processing allows for hardware failure contingency but comes with an increased development and deployment burden to effectively utilize this redundancy. Recoverability when failures are, inevitably, encountered on either a hardware or software level is also a requirement of the processing system as a whole. We will consider the following to be requirements of continuous processing designed to provide close-to-live feedback during data acquisition.

(i) *Modularity*: components should be sensibly separated to benefit code readability and maintainability. In the case of processing, components should act on the smallest coherent unit of input data to allow a simple path to scalability, among other benefits.

(ii) *Recoverability*: in the event of a failure the processing system should retain sufficient information about the failed processing to be able to reinject the request without any other impacts on the processing. This reinjection should be controllable by the maintainers of the system, as they may wish to postpone it while investigating causes of the failure.

(iii) *Extensibility*: as automation of processing develops it will be necessary to make extensions in a manageable way. Modularity is key to this goal, as the addition of new components should be achievable without the need to make major modifications to existing components.

(iv) *Resilience*: individual processing failures should not affect the processing of logically independent data.

(v) *Scalability*: to be able to scale the level of hardware resource leveraged against a particular processing problem dynamically to allow for different patterns of data collection. Ideally, this scaling has a close-to-linear relationship to the quantity of available hardware to simplify the understanding of future resource requirements under anticipated changes to data-collection rates.

(vi) *Monitoring*: for highly distributed workloads there must be centralized monitoring systems from which to debug potentially highly interconnected and complex processing failures across all components. There is also the opportunity to set up alerting systems so that minor failures can be noticed and dealt with on timescales that are appropriate to typical data collections.

As an example, if motion correction fails on a single movie in a tilt series due to a transient network filesystem outage this should not stop the ability to process that tilt series once the filesystem is recovered, nor should it affect the processing of other tilt series that are unaffected by the outage.

Processing must obviously be performed by software domain-specific to cryoEM, but processing pipelines that utilize these tools consistent with the above requirements may make use of the wide range of modern technologies designed to address some of these issues.

We stress here the importance of not just scalability when introducing new hardware but also in a dynamic form which can load-balance across existing resource. This allows methods of data acquisition that provide variable rates on different data domains to be accounted for. For example, the use of beam shift (Bouvette *et al.*, 2021 ▸; Eisenstein *et al.*, 2023 ▸), as opposed to stage movement, in tomographic data collection facilitates increased acquisition speeds, which poses two main resource challenges for live processing. One is simply that the needs for a given data collection are typically increased, with potentially hundreds of tilt series being collected over the course of two days. The other is the temporal profile of the collection. Without beam shift, tilt series would be expected to be collected in sequence, allowing time for reconstruction while the next tilt series is acquired. In beam-shift schemes a number of movies are collected at different positions on the grid using beam shift before the tilt angle of the stage is changed. This means that a large number of tilt series may be completed within a time window that is small compared with that associated with a tilt-series collection. If the processing is to remain live then this necessitates a relatively large application of computational resource at regular intervals to the specific task of reconstruction. The processing system should be able to scale reconstruction processing up and down to match this collection pattern.

## 4. Technologies

There are a range of standard tools developed for enterprise applications that aim to address the requirements raised in the previous section. Cloud infrastructure platforms offer environments for the deployment of a wide variety of such applications with resilience and scalability in mind. They also encourage modularity in the form of service-based architectures, with an emphasis on containerization of these services to allow deployment in a system-independent manner.

One such system, and that deployed on premises at Diamond Light Source (DLS), as well as other large-scale laboratories, is Kubernetes. Kubernetes is a container orchestration system. It manages services according to configured replica sets which are automatically maintained given sufficient hardware resources. This handles the resilience of processing components, with processing services that are running on failing hardware being replaced automatically. There are also tools allowing dynamic changes to these replica sets based on a variety of metrics.

In principle, the containerized services are deployable on the off-premises clusters of cloud service providers. The greatest difficulties arise in the early stages of processing, where rapid processing is required but the data rates are potentially large. The data transfer to an off-premises system then becomes the major bottleneck in the processing system, as well as a large source of cost. Work on cloud-provisioned cryoEM processing is typically focused on processing under the assumption that the full data set to be processed is available at processing time (White & Skjerven, 2022 ▸). Downstream processing steps are good targets for the utilization of off-premises resources, as the processing latency is less critical and the time taken in manipulation of the input data volume is often relatively small when compared with the algorithm time. However, on-premises systems are more suitable for live processing, where the priority is to minimize the time for results to be produced.

With anything other than very simple processing pipelines deployed in a distributed system, there will be a need for communication between components. Message brokers such as RabbitMQ (https://www.rabbitmq.com/) route messages through exchanges onto queues, from which they can be consumed. Messages can be programmatically acknowledged, with unacknowledged messages being requeued by the broker after a timeout is reached. This means that unexpected failures do not result in a loss of information. RabbitMQ forms the basis of Zocalo, as deployed at DLS, which provides a framework for developing services that consume from the message queues, with the services able to generate further messages along routes predefined in DAGs encoded in Zocalo ‘recipes’.

## 5. Implementation

In order to implement the processing graph, as described in Section 2.1, according to the principles outlined in Section 3, we will map these abstractions to the technologies considered in Section 4. The vertices in the processing graph naturally map to the services which perform the processing relevant to that vertex. The edges need to function as a communication layer between services, with information produced in one vertex fed as input to others. For this purpose we use messages sent via a RabbitMQ broker.

The services need to listen to a RabbitMQ queue for processing requests. We use the functionality of Zocalo to implement these services. The use of messaging queues provides a direct measure of the state of the various stages of a processing pipeline. Latency at any particular vertex will manifest as an increasing number of queued messages. The number of queued messages can be used as a metric to trigger the horizontal scaling of service instances that consume from that queue, allowing the processing system to dynamically react to demand and balance resources across processing components. For this, we use the event-driven autoscaler KEDA (https://keda.sh/). KEDA is a component which can be added to a Kubernetes cluster to provide dynamic scaling of replica sets according to a range of events from a number of widely used software components, including RabbitMQ.

The tomography pipeline is split into two distinct processing subgraphs (mapping to ‘recipes’ in the language of Zocalo) operating on different input domains. The first is the preprocessing pipeline, which performs motion correction and CTF estimation for each movie in the data collection. The second is the reconstruction pipeline, which runs tilt-series alignment and tomogram reconstruction. The preprocessing pipeline needs to be triggered as soon as possible after the transfer of each movie to the facility filesystem. The reconstruction pipeline needs to be triggered as soon as possible after all of the tilts in a series have been motion-corrected. The latter requires knowledge of which movies have been motion-corrected, which tilt series they belong to and when the acquisition of each tilt series has been completed. This is aggregated information that exists outside the context of individual preprocessing pipeline runs and requires a more centralized store of information.

The third panel of Fig. 1 ▸ depicts the communication between processing services, the RabbitMQ message broker and the centralized processing information store in the form of a PostgreSQL database, all deployed on Kubernetes.

In the implementation deployed at eBIC, the reconstruction and tomogram denoising components are targeted for the use of infrastructure provided by the STFC IRIS project. This is computational resource outside the DLS facility system in the Rutherford Appleton Laboratory (RAL) cloud. Once local GPU resources are saturated (*i.e.* all locally available resources are in concurrent use), services that perform submission to IRIS resources are spun up, consuming from the same queue as the local implementation of the service. This can then be performed in an automated manner that is completely hidden from the user while allowing a sensible balancing of resource usage.

Table 1 ▸ provides a breakdown of the services deployed at DLS as part of the eBIC cryoET processing pipeline. In addition to these services, we run a coordinating server on DLS storage servers which is described further in Section 6. The values of maximum number of instances for each service are designed to allow a rapid reduction of processing backlogs that may be caused by temporary outages of upstream services; typical usage is lower.

## 6. Connecting data collection and processing

In a typical cryoEM setup, fraction data are written to a server connected to the acquiring detector. Metadata from the acquisition software are written to a separate control machine, which is likely to share a network connection with the detector server, opening the possibility of also writing the metadata directly to the detector server. Facility computational resources such as those at DLS are unlikely to be directly connected to these vendor-installed filesystems. A link must therefore exist between the microscope and detector system and the facility filesystem through a storage server with the appropriate filesystem mount. The disconnection of the acquisition software from the processing hardware can be overcome by having a client running on the detector system which will orchestrate and monitor file transfer to the facility storage server. Processing requests will then be sent over a network connection to software running on that storage server. This also allows facility monitoring of data collection and transfer. Continuous transfer with removal of source fractions is important in cases where the available local storage on the detector server is limited to a few terabytes, in which case lack of disk space can be a barrier to unattended data collection.

The server component can connect over the network to the facility laboratory information-management system (LIMS; ISPyB in the case of DLS; Delagenière *et al.*, 2011 ▸) and any services required for data analysis. Processing can be requested at the point of transfer, reducing processing latency and avoiding the need for file watchers on the facility filesystem. The individual requests can also produce records allowing recoverability on the smallest scale. The client–server architecture may also act as an aggregation point for the information needed at points where the data domain on which processing steps act changes, as it has visibility over the whole processing workflow. In the tomography pipeline this point is when individual motion-corrected micrographs are stacked together into tilt series, but similar points occur in other processing pipelines. The aggregation can be achieved either in the memory of the client component or in a more sophisticated data-management system such as a database to which the server can make connections. At DLS such a client–server architecture, called *Murfey*, is currently under active development, not just for the tomography pipeline described here but also the more complex case of a single-particle analysis (SPA) pipeline. *Murfey* uses a PostgreSQL database to store data relevant to the coordination of pipelines such as which movies belong to which tilt series, whether the tilt series has been fully acquired and which movies have been motion-corrected.

## 7. Visualization

For the monitoring of data acquisition via processed data it is necessary to have a simple visualization tool which can display up-to-date information. At a facility this is combined with the needs of data security through authorization and authentication. The ISPyB schema contains tables designed to store cryoEM processing outputs for both SPA experiments and cryoET. At DLS, the system described above is also used to make inserts into ISPyB following the successful completion of processing. This uses a service dedicated to performing these operations.

There are a number of web frontends available for ISPyB with cryoEM visualization for SPA. When extending to cryoET the options were to extend an existing frontend, SynchWeb (Fisher *et al.*, 2015 ▸) in the case of DLS, or develop a new one using more modern technologies. This latter option fits with the modularity principle outlined above: a separation of the visualization of cryoEM processing results from the other services offered by the monolithic SynchWeb application such as macromolecular X-ray crystallography processing visualization and sample shipping and tracking.

**Figure 2:**
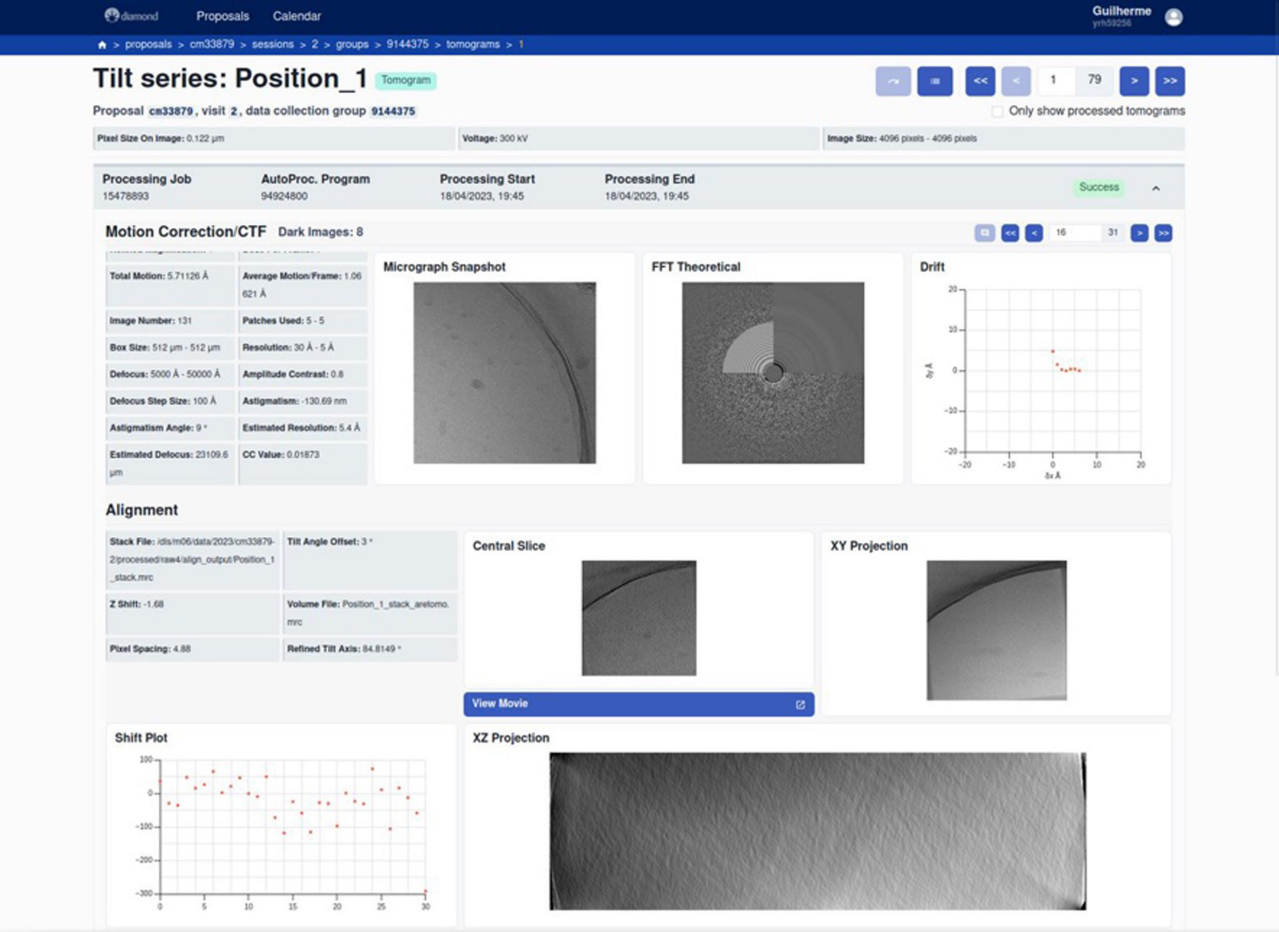
*PATo* primary tomography processing display. Tilt series can be paged through and the tomogram itself opened to pan along the *z* axis.

A new web application (*PATo*) was developed for the visualization of cryoEM data-processing results (both SPA and cryoET) using a Python backend built on the FastAPI (https://fastapi.tiangolo.com/) framework and a React (https://react.dev/) frontend. For cryoET, motion-corrected micrographs and micrograph power spectra are displayed, collected by tilt series (Fig. 2 ▸). Once a tomogram has been reconstructed the central slice and projections are displayed with the option to open the tomogram and pan through the 3D volume along the *z* axis. If the tomogram has been denoised then the denoised volume is displayed alongside the raw tomogram.

## 8. Conclusions

We have reported on the deployment of a relatively simple cryoET processing pipeline, ending in tomogram reconstruction and denoising, on modern computing architecture, in particular as services on a Kubernetes cluster. This has demonstrated an ability to provide fast feedback from processing during data collection at eBIC with responsive dynamic scaling capabilities. This is particularly relevant at scale, either in terms of the number of data collections or throughput, with the latter being increased by the relatively recent application of beam-shift techniques to cryoET data acquisition. In order to meet the requirements of robust live processing, we have made use of distributed systems, and some familiarity with such systems is, unfortunately, necessary to deploy and maintain such a system. Processing results are stored in an instance of the ISPyB LIMS (Delagenière *et al.*, 2011 ▸) and a new web application has been developed for visualization, allowing users to monitor the live processing of their data collections and make acquisition adjustments as necessary.

The principles outlined here for tomography processing are applicable to other pipelines, for example the considerably more complicated SPA processing pipeline used at eBIC. This requires additional services and coordination of different processing stages. This work has been completed and deployed at eBIC and will be described in detail elsewhere. Some statistics regarding the usage of the cryoET deployment to support the user and internal research programmes at eBIC during 2023 are given in Appendix *A* [App appa].

## 9. Source-code availability

*Murfey*: https://github.com/DiamondLightSource/python-murfey.

*PATo* frontend: https://github.com/DiamondLightSource/pato-frontend.

*PATo* backend: https://github.com/DiamondLightSource/pato-backend.

Processing services and partial configuration: https://github.com/DiamondLightSource/cryoem-services.

## Figures and Tables

**Figure 3:**
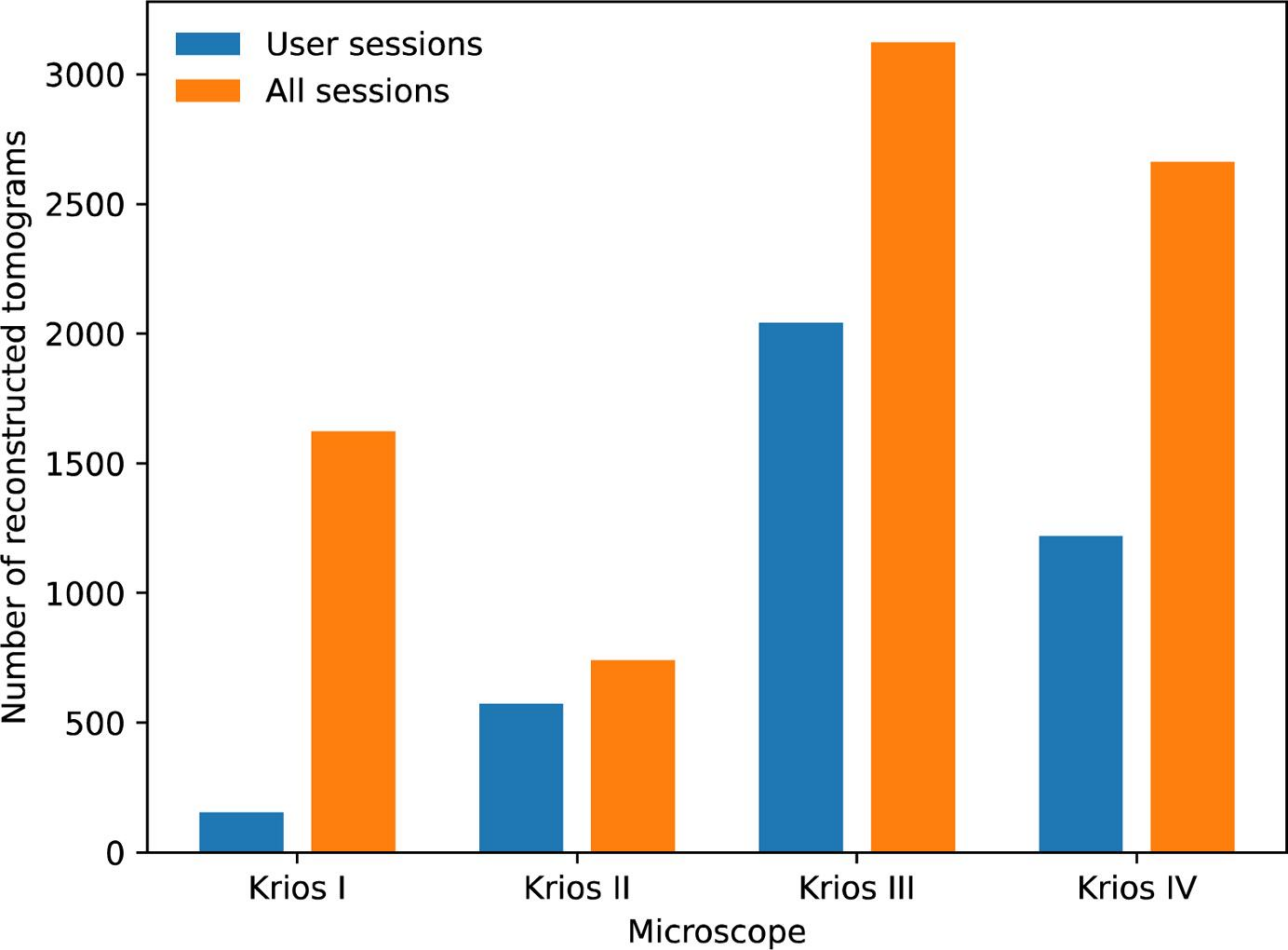
Tomography pipeline usage statistics at eBIC for 2023, displaying the number of tomograms reconstructed by automated systems. The figure is broken down across the four Krios microscopes primarily used for academic work. ‘All sessions’ refers to the sum of user, internal research and commissioning sessions. There were a total of 8146 reconstructed tomograms.

**Table 1:** Processing components deployed for the cryoET pipeline at eBIC along with approximate resource requirements and limits These requirements are designed to be able to accommodate live data analysis on four Titan Krios microscopes. Note that Kubernetes resource requirements can be specified in fractions of CPU cores, in which case the CPU clock cycles are divided between applications. A100, V100 or P100 GPUs are used as the GPU resource, but lower CUDA core-count GPUs would be adequate. Some motion-correction services submit to a separate HPC cluster operating a Slurm scheduler. A larger number of motion-correction services than necessary are allowed for the pipeline to be able to deal with backlogs. Typical usage only sees approximately four service instances in use.

Service component	Maximum No. of instances	Minimum requested CPU cores (per instance)	Maximum CPU cores (per instance)	GPU resources (per instance)
Motion-correction service (*MotionCor*2)	8	0.5	1	1 GPU, V100 or P100
CTF-estimation service (*CTFFind*4)	4	0.25	1	None
Reconstruction service (*AreTomo*)	4	0.5	1	1 GPU, V100 or A100
Tomogram denoising (*Topaz*)	2	0.5	1	1 GPU, V100 or A100
ISPyB connector service	4	0.25	1	None
Images service (for thumbnail creation and data-format conversion)	4	0.25	1	None
Dispatcher (workflow-triggering service)	2	0.25	1	None
RabbitMQ server	1	0.5	1	None
PostgreSQL database servers (used by *Murfey*)†	3	0.5	2	None
PgPool PostgreSQL middleware†	2	1	2	None

† Part of a standard high-availability deployment. The relevant Helm chart is available at https://artifacthub.io/packages/helm/bitnami/postgresql-ha.

## Appendix A Pipeline utilization at eBIC

The automated tomography pipeline has been in use at eBIC throughout 2023. It was designed to accommodate increases in the number of tomography sessions and the increasing data-collection rates in tomography. On user visits the tomography pipeline has been used to reconstruct 3987 tomograms, with a total of 10 585 across all sessions. The breakdown across the four Titan Krios microscopes for academic use at eBIC is shown in Fig. 3 ▸.

To reduce compute time and storage requirements we potentially perform binning at two stages in the pipeline. During motion correction micrographs are binned to the size of the physical pixel array of the detector (*i.e.* a binning factor of 2 is applied to any data collected in superresolution). Secondly, a binning factor of 4 is applied during reconstruction. Motion-corrected stacks that act as input to *AreTomo* are typically approximately 2.5 GB per tilt series and the resulting tomograms are approximately 1.2 GB. The raw data volumes vary considerably with exposure time and format (where the difference arises from compression schemes or lack thereof). However, if sufficiently compressed (*i.e.* using LZW-compressed TIFF) the raw tilt-series data will not exceed that of the motion-corrected stack, as the latter is in uncompressed MRC format. This is performed for simple compatibility with a range of cryoEM processing software.
